# A risk factor analysis of complications after surgery for vulvar cancer

**DOI:** 10.1007/s00404-020-05949-w

**Published:** 2021-01-09

**Authors:** Georgios Gitas, L. Proppe, S. Baum, M. Kruggel, A. Rody, D. Tsolakidis, D. Zouzoulas, A. S. Laganà, V. Guenther, D. Freytag, I. Alkatout

**Affiliations:** 1 Department of Obstetrics and Gynecology, University Hospital of Schleswig Holstein, Campus Luebeck, Ratzeburger Allee 160, Haus A, 23538 Luebeck, Germany; 2 First Department of Obstetrics and Gynecology, Aristotle University of Thessaloniki, Thessaloniki, Greece; 3 Department of Obstetrics and Gynecology, Filippo Del Ponte Hospital, University of Insubria, Varese, Italy; 4 Department of Obstetrics and Gynecology, University Hospital of Schleswig Holstein, Campus Kiel, Kiel, Germany

**Keywords:** Vulvar cancer, Postoperative complications, Lymphedema, Wound dehiscence, Resection margin

## Abstract

**Introduction:**

Despite the less frequent use of surgery in patients with vulvar cancer, the high rates of postoperative complications are still a matter of concern. The aim of the present study was to identify risk factors that influence postoperative complications rates in vulvar cancer and identify specific clinical parameters that may influence their incidence.

**Materials:**

Patients who underwent curative-intent surgery for squamous cell carcinoma of the vulva from 2003 to 2018 were selected. All patient characteristics were analyzed as risk factors for the development of postoperative lymphocele, lymphedema, and wound dehiscence. The patients were followed up for 2 years postoperatively.

**Results:**

The investigation comprised 121 patients, of whom 18.1% developed wound dehiscence, 17.7% a lymphocele, and 20.4% lymphedema. We found no significant evidence of an association between patient’s characteristics and postoperative complications. The depth of tumor invasion and the appearance of lymph-node metastasis were significantly associated with postoperative complications. Free resection margins of 5 mm or more were associated with a reduced risk of postoperative complications compared to resection margins less than 5 mm. No complications were encountered after sentinel node biopsy (SNB). Complication rates were associated with inguinofemoral lymphadenectomy, but not with the extent of lymphadenectomy. The development of a lymphocele or wound dehiscence may be correlated with the development of long-term lymphedema.

**Conclusion:**

FIGO stage at diagnosis influences the risk of postoperative complications. The use of SNB minimized postoperative complications. Correlations between the free microscopic resection margin distance and the risk of postoperative wound dehiscence must be investigated further.

## Introduction

After cancers of the uterine corpus, ovaries, and cervix, vulvar cancer is the fourth most common gynecologic cancer in women; accounting for 5% of all malignancies of the female genital tract [1]. The most common histological type is squamous cell carcinoma of the vulva (95%), followed by melanoma, sarcoma, and basalioma [1]. For a long period of time, the peak incidence of vulvar cancer was between the ages of 65 and 75 years. However, in the last few decades, a significant number of women have developed vulvar cancer at a younger age (35–65 years) because of the increasing numbers of human papillomavirus (HPV)-related cases of this malignant disease [2].

In the 1980s, surgical treatment of vulvar cancer consisted of radical en bloc resection of the tumor with bilateral inguinofemoral lymphadenectomy (IFLND), as described by Rutledge. This concept has been rejected because of high complication rates [3]. Currently, the standard surgical procedure for the treatment of vulvar cancer is the triple incision technique (separate incisions for groin node dissection). Under specific requirements, such as a unifocal tumor less than 4 cm in size and unsuspicious lymph nodes, the surgeon performs sentinel lymph-node (SLN) mapping of the inguinofemoral lymph nodes [4]. This approach proved to be very effective. Nevertheless, approximately one-half of patients with vulvar cancer need to undergo an IFLND [5].

Of all gynecological cancers, patients with vulvar cancer have the lowest quality of life score [6]. The most severe and common postoperative complications of vulvar cancer surgery are lymphedema, lymphocele, and wound dehiscence. En bloc surgery raised complication rates after IFLND to 85% and wound dehiscence to 70–90% [7]. The establishment of the triple incision technique as the gold standard and the use of the sentinel node technique in less advanced stages of the disease reduced postoperative complication rates significantly. However, despite advancements in surgery and innovation in available devices [8, 9], complication rates remain high in patients with vulvar cancer. Currently, 17–39% of patients experience wound dehiscence, 7–40% a lymphocele, and 14–48.8% lymphedema [10]. All of these complications are associated with high morbidity rates and poor quality of life [10].

The aim of the present study was to identify risk factors that influence the incidence of the most frequent and severe postoperative complications of vulvar cancer. The risk factors could also be viewed as prognostic factors affecting the patients’ quality of life and mortality. To our knowledge, this is the first study addressing the correlation between the free resection margin distance and wound dehiscence.

## Materials and methods

A retrospective study was carried out at the department of obstetrics and gynecology, University of Luebeck, from 2003 to 2018. We identified women who had undergone surgery for squamous cell carcinoma of the vulva. The study was approved by the local ethics committee. The patients’ medical history, histology, and postoperative data were pseudonymized and recorded. Patients with inoperable vulva cancer or those who had undergone pelvic exenteration were excluded. The patients’ pathological data are listed in Tables 1 and 2.

**Table 1:** Characteristics of the patients who underwent surgery for vulvar cancer

Patient characteristics	
Age (years)	69.4 ± 15.3
BMI (kg/m^2^)	27.5 ± 6.1
Smoking (*n*)	36
Lichen sclerosis (*n*)	21
BMI > 30 (kg/m^2^)	38
Diabetes mellitus (*n*)	23
Arterial hypertension (*n*)	67
History of vulvar intraepithelial neoplasia (VIN)	55
VIN I	3
VIN II	6
VIN III	46
Location of the tumor (*n*)	
Right side	29
Left side	27
Both sides	60
Undefined	5
Clitoris	25
Labia majora	55
Labia minora	51
Sub-clitoral	30
Posterior commissure	14
ECOG score (*n*)	
0	36
1	44
2	25
3	14
4	0
FIGO (*n*)	
Ia	16
Ib	73
II	6
IIIa	7
IIIb	8
IIIc	9
IVa	0
IVb	2

**Table 2:** Intraoperative and histological characteristics of patients

Details of operation and histology	
Duration of surgery (average in minutes)	119.4 ± 84.3
Duration of postoperative hospital stay (average in days)	9.7 ± 9.1
Method of surgery (*n*)	
Vulvectomy	19
Hemivulvectomy	69
Local excision	33
Lymphadenectomy (*n*)	
Yes	95
No	26
Lymph-node excision (average *n*)	11.2 ± 11
Patients with lymph-node metastasis (*n*)	27
Lymph node metastasis by sentinel	1 ± 0
Lymph node metastasis by IFLND	3.24 ± 2.02

Type of operation	Number of operations	Lymph-node metastasis (*n*)
IFLND	41	17
SNB	24	0
IFLND after SNB	30	7
Pelvic LNE (primary)	1	1
Pelvic LNE (secondary)	8	8

Details of operation and histology	
Clavien–Dindo (*n*)	
0 and 1	78
2	22
3a and 3b	21
4 and 5	0
Histology	
Unifocal	96
Multifocal	25
Tumor spread grading (*n*)	
I	16
II	77
III	15
Lymphatic invasion (*n*)	12
Venous invasion (*n*)	2
Perineural invasion (*n*)	6
Tumor size (diameter in mm)	29.2 ± 22.3
Depth of tumor invasion (mm)	5.8 ± 6.9
Tumor resection margin distance (mm)	
0–3 mm	28
3–5 mm	24
5–8 mm	35
> 8 mm	17

	< 5 mm	≥ 5 mm	Total (104 patients)	*p *value
FIGO stage and tumor resection margin distance				
FIGO I	37	38	75	n.s
FIGO II	2	3	5	n.s
FIGO III	12	10	22	n.s
FIGO IV	1	1	2	n.s

The FIGO system was used to categorize the disease. The patients then received appropriate surgery consisting of vulvar excision (local excision, hemivulvectomy, vulvectomy, radical vulvectomy, and vulvectomy with plastic reconstruction), and further operative staging such as lymphadenectomy if necessary.

According to our clinical protocols which concur with the German guidelines [11], patients were given single-shot intravenous antibiotic prophylaxis intraoperatively. Depending on the location of the tumor, we performed wide local excision or vulvectomy (partial, total, or radical) with or without flap reconstruction, with a free clinical margin of 1–2 cm. No drain was placed in the vulvar region. The triple incision technique was used in all patients. A second resection was considered if the resection margin in healthy tissue was 3 mm or less.

SLN mapping of inguinal lymph nodes was performed with technetium-99 (99mTc) when the tumor was less than 4 cm in size, unifocal, and with unremarkable lymph nodes on ultrasound investigation. The IFLND included the removal of both, superficial and deep inguinofemoral lymph nodes. We always tried to preserve the saphenous vein if possible. A sartorius transposition was not performed. A vacuum drain was placed in each groin after sentinel or radical lymphadenectomy. The drains were removed postoperatively, after at least 2 days, when the total output was less than 30 ml/24 h. Postoperative complications were classified according to the Clavien–Dindo scale [12].

Complications (lymphocele, lymphedema, and wound dehiscence) were recorded on two occasions: until 1 month after the operation, and at least once over a follow-up period of 2 years. All patients received a follow-up letter. Lymphoceles and lymphedema were evaluated in patients who had undergone surgery in the groin with SLN biopsy or IFLND as part of their treatment of vulvar cancer. A subgroup analysis (≤ 10 lymph nodes removed vs. > 10 lymph nodes removed during IFLND) was performed according to Courtney-Brooks et al. [13], to determine whether the extent of IFLND might be a risk factor for the development of postoperative lymphocele or lymphedema. Wound dehiscence was investigated only in the vulvar region.

We examined correlations between lymphocele, lymphedema, and wound dehiscence on one hand, and the above-mentioned patient characteristics on the other. A subgroup analysis was performed in regard to the microscopic resection margin distance (pathological or free resections margin between 0–3 mm, 3–5 mm, and 5 mm or more) and the risk of wound dehiscence. Patients who underwent repeat surgery or radiotherapy because of pathological resection margins were excluded from the subgroup analysis. A further analysis was performed to compare complication rates after SNB or IFLND. Moreover, the correlation between early (lymphedema or wound dehiscence) and late complications (lymphedema) was examined.

Statistical analyses were performed using the free ANACONDA/Python software (*Anaconda Software Distribution*. Computer software. Version 2–2.4.0. Anaconda, Nov. 2016. Web. < https://anaconda.com >.), Version 3.7, including the packages pandas, numpy, scipy, pingouin, and researchpy [14]. Depending on the scaling and distribution of the variables considered, either a Chi-square test, a Mann–Whitney *U *test, or a one-way-ANOVA was performed. *p* values less than or equal to 0.05 were considered statistically significant. When a one-way-ANOVA or a Chi-square test was performed, a post hoc test (Tukey’s post hoc test) was conducted to determine those variables or combinations of variables responsible for the variation of the target variable.

## Results

The study comprised 121 patients with squamous cell carcinoma of the vulva. Patient characteristics are listed in Table 1. BMI and FIGO stages were not significantly correlated to surgical complications.

The majority of patients (95cases) underwent an additional inguinal lymphadenectomy: SNB alone was performed in 24 patients, a primary IFLND in 41, and both procedures in 30 patients. Further intraoperative and histological details are shown in Table 2. Twenty-two of 121 (18.1%) patients developed wound dehiscence and 17 out of 96 (17.7%) developed a lymphocele. Fourteen patients died during the follow-up period and were excluded from further analysis. Ten of 49 patients (20.4%) experienced chronic lymphedema a few years after the operation.

No significant association is calculated between patients’ characteristics and the development of postoperative complications (Table 3). The duration of treatment in the hospital and the duration of surgery (*p* < 0.001) were significantly longer in patients who experienced wound dehiscence (*p* < 0.001).

**Table 3:** Analysis of patient’s characteristics and postoperative complications

Complications Characteristics	Lymphocele (*n* = 17)	Lymphedema (*n* = 10)	Wound breakdown (*n* = 22)	Total (*n* = 121)	*p *value
BMI (kg/m^2^)	28.4 ± 6.5	26.7 ± 5.5	30.5 ± 7.4	27.5 ± 6.1	n.s
Age (years)	75.6 ± 10.0	70.9 ± 12.9	74.4 ± 11.4	69.4 ± 15.3	n.s
Nicotine abuse (*n*)	4 (23.5%)	1 (10%)	1 (4.5%)	36 (29.8%)	
Lichen sclerosis (*n*)	1 (5.9%)	0	2 (9.1%)	21 (17.4%)	
Arterial hypertension (*n*)	14 (82.4%)	6 (60%)	20 (90.9%)	67 (55.4%)	
VIN III (*n*)	4 (23.5%)	3 (30%)	4 (18.2%)	46 (38%)	
Number of resected lymph nodes	17.5 ± 15*	20.6 ± 20*	21–5 ± 17.7*	13.7 ± 11.6	< 0.01
Duration of surgery (minutes)	170.6 ± 77.2	180.2 ± 84.8	196.5 ± 121.5*	119.4 ± 84.7	0.01
Multifocal tumor (*n*)	3 (17.6%)	3 (30%)	7 (31.8%)	25 (20.7%)	
Lymphatic invasion (*n*)	2 (11.8%)	0	5 (22.7%)	12 (9.9%)	
Average depth of invasion (mm)	6.9 ± 5.8	10.5 ± 12.8	11.0 ± 10.3*	5.7 ± 7.0	0.01
Resection margin (mm)	5.2 ± 3.9	4.8 ± 4.6	3.6 ± 3.0	4.8 ± 3.4	
Radiation therapy (*n*)	4 (23.5%)	6 (60%)	9 (40.9%)	19 (15.7%)	
Chemotherapy (*n*)	0	0	2 (9.1%)	3 (2.5%)	
ECOG (average)	1.5	1.2	1.7	1.1	
Duration of treatment in the hospital (days)	12.5 ± 6.7	12.6 ± 8.1	19.6 ± 15.2	9.7 ± 9.1	
FIGO I and II (*n*)	11 (64.7%)	4 (40%)	12 (54.5%)	95 (78.5%)	
FIGO III and IV (*n*)	6 (35.3%)	6 (60%)	10 (45.5%)	26 (21.5%)	

*p *values calculated via ANOVA

*Significant differences

The depth of tumor invasion is significantly associated with the wound dehiscence (*p* = 0.001). Patients with lymph-node metastases have a significantly higher risk of developing lymphocele, lymphedema, or wound dehiscence (*p* = 0.005) (Table 3). Moreover, it seems that patients with advanced tumor stages (FIGO III, IV) tend to develop more often postoperative complications compared to those with a low tumor stage (FIGO I, II, Table 3).

**Fig. 1:**
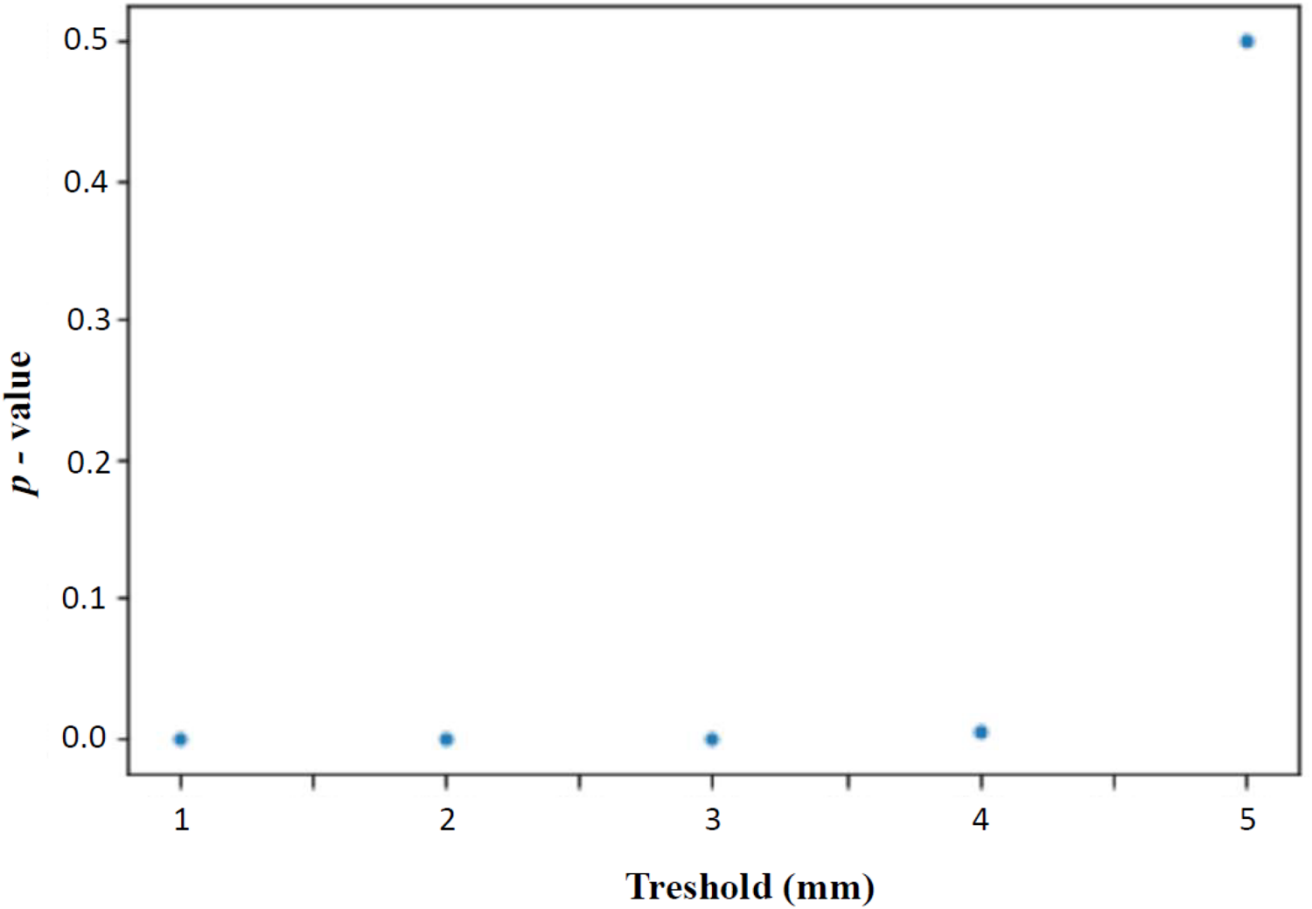
Association between microscopic free resection margin in millimeters (mm) after vulvectomy and the risk of postoperative complications. Patients with a free resection margin of 5 mm do not have a significant increased risk developing postoperative complications compared to those with a free resection margin under 5 mm

To determine a threshold for a resection margin at lowest risk for developing postoperative complications such as lymphedema, lymphocele, or wound-healing disorder, we performed an exploratory analysis. As shown in Fig. 1, patients with a free resection margin of less than 5 mm do have a significantly increased risk (*p* ≤ 0.05) to develop postoperative complications compared to those with a free resection margin of 5 mm or more. Patients categorized into those with resection margin 0–5 mm and those < 5 mm were comparable concerning FIGO stage (Table 2).

The subgroup analysis of complication rates after SNB or IFLND revealed that IFLND is associated with a significantly higher rate of postoperative lymphocele (*p* < 0.001), lymphedema (*p* < 0.001), and wound dehiscence (*p* < 0.001) compared to SNB (Table 4). Patients with postoperative complications had significantly more resected lymph nodes compared to those who underwent a lymphadenectomy but had no postoperative complications such as lymphocele or wound-healing disorder (*p* < 0.01). We could not determine a threshold concerning the amount of resected lymph nodes which might indicate less postoperative complications.

**Table 4:** Association of inguinofemoral lymphadenectomy and sentinel node biopsy with postoperative complications

	SNB (24 patients)	IFLND (71 patients)	Total (95 patients)	*p *value
Lymphocele	0	16	16	< 0.001
Lymphedema	0	10	10	< 0.001
Wound-healing disorder	1	19	20	< 0.001

*IFLND* inguinofemoral lymphadenectomy, *SNB* sentinel node biopsy

A further analysis revealed that the development of early complications (lymphocele or wound dehiscence) might be correlated with the development of long-term lymphedema. Of patients who developed long-term lymphedema, 5/10 experienced wound dehiscence postoperatively and 6/10 developed a lymphocele. Four of the patients without early complications developed long-term lymphedema (6.4%), whereas the possibility was higher for the patients with early complications (18.2%).

## Discussion

The present investigation revealed specific prognostic factors that may influence postoperative complication rates in patients with vulvar cancer. Free resection margins of 5 mm or more were not significantly associated with the risk of postoperative wound dehiscence compared to those with resection margins less than 5 mm. The use of SLB minimized postoperative complications. Furthermore, complication rates were associated with IFLND or lymph-node metastasis, but the results did not allow to determine a threshold concerning the extent of lymphadenectomy and the risk of postoperative complications. Moreover, the development of lymphocele or wound dehiscence was correlated with long-term lymphedema.

Our analysis revealed that 17.7% of patients developed a lymphocele, 18.1% wound dehiscence, and 20.4% chronic lymphedema. These data concur with the low complication rates reported in the published literature (lymphocele 14–48.8%, wound dehiscence 17–39%, and lymphedema 7–40%) [10]. All patients were subject to the same high-quality standards of treatment, including experienced surgeons, wound care, the triple incision technique with the use of sentinel biopsy, prophylactic intraoperative antibiotics, and drains. It should be noted that we still lack large randomized trials concerning the appropriate treatment strategy for vulvar cancer.

Our analysis revealed no preoperative risk factors for the development of postoperative complications. We registered no significant association between age, BMI, and obesity (BMI > 30 kg/m^2^) on one hand, and complications such as postoperative lymphocele, wound dehiscence, and chronic lymphedema on the other. Our data concur with those reported by Gaarenstroom et al. who found no association between advanced age and the occurrence of complications in 187 women with vulvar cancer [15]. In an investigation comprising 56 patients who underwent IFLND, Walker et al. mentioned that age and BMI were not associated with wound dehiscence [16]. On the other hand, age and BMI were reported as independent risk factors for complications after surgery for vulvar cancer [5, 17]. In a study consisting of 99 patients with vulvar cancer, Cierik et al. registered a higher risk of postoperative wound dehiscence in patients older than 65 years and obese persons (BMI > 30 kg/m^2^), whereas younger age was found to be an independent risk factor for postoperative lymphedema [17]. The interpretation of these results was based on the hypothesis that young women were more active preoperatively than older ones, are suddenly immobilized after the operation, and therefore experience lymphedema more frequently. The above inhomogeneous data reveal that preoperative risk factors are still a debated issue in patients with vulvar cancer.

Our study revealed that a free microscopic resection margin of more than 5 mm might be used to reduce the risk of postoperative wound dehiscence. Cancer cell proliferation is known to cause retraction and scars in the neighboring normal tissue. Furthermore, the wound environment and the high cytokine concentration may cause cancer cells from other locations to move into the wound area and lead to wound dehiscence [18]. Using immunohistochemical methods, Stanczyk et al. examined the influence of cancer on the wound-healing process after the excision of metastatic liver tumor [19]. The authors found that poor scar tissue formation, the migration of cancer cells to the wound, and the poor proliferation of inflammatory cells impair the healing process. The above hypothesis might explain our results. A large free resection margin may reduce the migration of cancer cells to the wound. The association between the free microscopic resection margin distance, local recurrence, and long-term outcomes is a strongly debated issue. The German guidelines recommend a free microscopic resection margin of 3 mm or more, whereas other studies mention a distance of 10 mm [20]. The oncological outcome is always the foremost parameter, but patients with vulvar cancer experience high complication rates which affect their morbidity, quality of life, as well as mortality [10, 21]. Thus, a potential reduction of complication rates by a free resection margin of more than 5 mm would be an attractive option. We recommend that the type of incision should be adapted to the individual patient, taking the location of the tumor and risk factors for complications into account. However, this thesis must be investigated in future studies.

The mean number of nodes dissected in our study was 11.2 ± 11. Patients with postoperative complications had more resected lymph nodes; however, the dissection of a large number of lymph nodes (more than 10) during IFLND was not significantly associated with a higher rate of postoperative lymphocele or lymphedema. The thesis that fewer lymph nodes may drain less lymphatic fluid and thus lead to the above-mentioned complications was not confirmed in our study. In an investigation comprising 164 patients who underwent IFLND for vulvar cancer, Hinten et al. discussed that a higher average number of dissected lymph nodes are not a risk factor for lymphedema [5]. The published literature in this regard is limited. The majority of studies examined the prognostic impact of the number of dissected lymph nodes. We postulate that, apart from the number of dissected lymph nodes, the anatomical location of dissection may influence the results.

The GROINSS-V study [4], which is the largest published prospective trial comprising 259 patients who underwent SNB in early vulvar cancer using 99mTc and blue dye, revealed that the SLN procedure reduced postoperative morbidity rates significantly compared to IFLND. The rate of lower limb lymphedema was 1.9% in women who underwent SNB, and 25.2% in those who underwent IFLND [22]. We performed SLN only with 99mTc and registered similar data as those reported in the GROINSS-V study. We registered no case of lymphedema or lymphocele after SLN. In the present study, IFLND was significantly associated with higher rates of postoperative lymphocele (*p* < 0.001), lymphedema (*p* < 0.001), and wound dehiscence (*p* < 0.001). A lymphocele was seen in 22.5% of patients who underwent IFLND, lymphedema in 14.1%, and wound dehiscence in 26.8%. Regrettably, the majority of patients (74.7%) were not eligible for the SLN procedure because of suspicious lymph nodes or a tumor size in excess of 4 cm. The avoidance of IFLND by the use of SLN after neoadjuvant therapy in patients with lymph-node metastasis might be an important factor in reducing postoperative complication rates. Future scientific evidence in this field could lead us away from the notion of “everything or nothing at all” to the acceptance of “not much, but good”, as currently applied in breast surgery.

The duration of treatment in the hospital and the duration of surgery were significantly longer in patients with wound dehiscence; this may be associated with the complexity of the operation and the need for postoperative wound management. However, the prolonged duration of surgery is correlated with postoperative infection and inductively with wound dehiscence [23]. In a study comprising 234 patients who underwent surgery for vulvar cancer, Dorney et al. noted that prolonged hospitalization, comorbidities, radical vulvectomy, nodal assessment, and the initial length of hospital stay were associated with hospital readmission rates [24]. The risk of early removal of drains (lymphoceles, infection, and wound dehiscence) must be weighed against the disadvantages of a prolonged hospital stay (pulmonary complications and infection). Early discharge from the hospital with in situ drains may help to prevent these complications and must be investigated in future studies.

In a large investigation, Gaarenstroom et al. observed a significant correlation between early wound complications and the development of late complications such as lymphedema [15]. From our data, we also hypothesize that the development of early postoperative complications may induce long-term lymphedemas. 10 patients from those surveyed during the follow-up period, developed a lymphedema. Out of these, five cases of wound-healing disorder and six cases of lymphocele are known, indicating the possible relation between early and late surgical complications. The possibility of developed long-term lymphedema patients without early complications was only 6.4%. On the other hand, in two published studies [25, 26] comprising 64 and 100 patients, the long-term follow-up revealed no correlation between early and late complications. Similar data were reported in a recent investigation by Soliman et al. [27] consisting of 64 patients. However, the low statistical power of these studies due to their small sample sizes impair their statistical validity. Preventive measurements in patients at high risk of lymphedema [28], such as maintaining normal body weight/avoiding weight gain, and a supervised exercise regimen may reduce morbidity.

The main limitation of the present study is its retrospective nature. Moreover, our study is based on data from a single center and that may be a significant limitation, and thus, department protocols, resources, and consequently composition of catchment population are probable boundaries to the generalizability of our results. However, it was based on detailed clinical information and demographic data for all patients. The relatively large sample size concerning complications after surgery for vulvar cancers. To our knowledge, this is the first investigation addressing the correlation between the free resection margin distance and wound dehiscence.

## Conclusion

The present study revealed a low incidence of complications after surgery for vulvar cancer. The standard of therapy, such as wound care, the triple incision technique with the use of sentinel biopsy, the administration of prophylactic intraoperative antibiotics, and drains until the achievement of a drain output less than 30 ml/24 h, may have contributed to this fact. However, complications after surgery for vulvar cancer are still a matter of concern, because it affects the patients’ quality of life and the outcome of treatment. No significant risk factors were identified in the present study. We hypothesize that the occurrence of early complications might induce late lymphedemas. However, early complications may be viewed as a reason to initiate preventive measures. The use of SLB minimizes postoperative complications. Therefore, early detection of vulvar cancer by gynecologists remains the key factor to reduce complication rates. The present study yielded new data about the correlation between the free microscopic resection margin distance and postoperative complications such as wound dehiscence or disorders of the lymph drain. A resection margin of 5 mm or more may reduce the risk of postoperative complications, but this thesis has to be validated in further investigations.

## Data Availability

The datasets used and analyzed during the current study are available from the corresponding author on reasonable request.
